# DHA and 19,20-EDP induce lysosomal-proteolytic-dependent cytotoxicity through de novo ceramide production in H9c2 cells with a glycolytic profile

**DOI:** 10.1038/s41420-018-0090-1

**Published:** 2018-08-20

**Authors:** Tomoko Endo, Victor Samokhvalov, Ahmed M. Darwesh, Kevin M. W. Khey, Ahmed A. El-Sherbeni, Ayman O. S. El-Kadi, Takuji Machida, Masahiko Hirafuji, John M. Seubert

**Affiliations:** 1 Faculty of Pharmacy and Pharmaceutical Sciences, University of Alberta, Edmonton, AB Canada; 2 Department of Pharmacological Sciences, School of Pharmaceutical Sciences, Health Sciences University of Hokkaido, Hokkaido, Japan; 3 Department of Clinical Pharmacy, Faculty of Pharmacy, Tanta University, 8130 Tanta, Egypt; 4 Department of Pharmacology, Faculty of Medicine and Dentistry, University of Alberta, Edmonton, AB Canada

**Keywords:** Cell death, Biochemistry

## Abstract

Docosahexaenoic acid (DHA) and their CYP-derived metabolites, epoxydocosapentaenoic acids (EDPs), are important fatty acids obtained from dietary sources. While it is known that they have significant biological effects, which can differ between cell type and disease state, our understanding of how they work remains limited. Previously, we demonstrated that DHA and 19,20-EDP triggered pronounced cytotoxicity in H9c2 cells correlating with increased ceramide production. In this study, we examine whether DHA- and 19,20-EDP-induced cell death depends on the type of metabolism (glycolysis or OXPHOS). We cultivated H9c2 cells in distinct conditions that result in either glycolytic or oxidative metabolism. Our major findings suggest that DHA and its epoxy metabolite, 19,20-EDP, trigger cytotoxic effects toward H9c2 cells with a glycolytic metabolic profile. Cell death occurred through a mechanism involving activation of a lysosomal-proteolytic degradation pathway. Importantly, accumulation of ceramide played a critical role in the susceptibility of glycolytic H9c2 cells to cytotoxicity. Furthermore, our data suggest that an alteration in the cellular metabolic profile is a major factor determining the type and magnitude of cellular toxic response. Together, the novelty of this study demonstrates that DHA and 19,20-EDP induce cell death in H9c2 cells with a glycolytic metabolicwct 2 profile through a lysosomal-proteolytic mechanism.

## Introduction

Long chain n-3 polyunsaturated fatty acids (PUFAs) such as docosahexaenoic acid (DHA, C22:6n-3) and eicosapentaenoic acids (C20:5n-3) are important fatty acids obtained from dietary sources. These essential fatty acids are required components of phospholipid membranes and serve as precursors to numerous lipid mediators with various biological properties. Numerous studies report a positive effect of n-3 PUFAs toward the cardiovascular system, suggesting they reduce the risk of cardiovascular disease by protecting the heart and vasculature against injury, such as limiting cardiac arrhythmias, myocardial infarction and hypertension^1,2^. Overall, there is a growing body of evidence demonstrating that n-3 PUFA have significant biological effects depending upon the cell and disease; however, understanding exactly how n-3 PUFAs work remains unknown. In recent years, evidence indicate that there is a biological role for cytochrome *P*450 (CYP) epoxygenase metabolites of DHA^3,4^. CYP epoxygenases metabolize DHA into 6 regioisomeric epoxydocosapentaenoic acids (4,5-, 7,8-, 10,11-, 13,14-, 16,17- and 19,20-EDP), which may then undergo further metabolism by epoxide hydrolase enzymes to corresponding diols, dihydroxydocosapentaenoic acids^5^. EDPs have been suggested as the active mediators impacting cellular responses to injury and disease such as cytotoxicity, cardiovascular disease and cancer^5–9^. Among the regioisomeric metabolites of DHA, 19,20-EDP gained the most interest due to its pronounced biological effects in numerous aspects of cell biology^10^.

The reported biological effects of DHA and its metabolites, EDPs, appear to be dependent upon the specific cellular phenotype being studied. H9c2 myoblast cells are an immortalized cell line derived from ventricular tissue of the BDIX rat heart^11,12^. H9c2 cells are not fully differentiated until cultured in media with low serum, which triggers differentiating from mono-nucleated myoblasts to a skeletal muscle phenotype or the addition of all-*trans* retinoic acid to 1% serum media that induces adult cardiac muscle phenotype^13,14^. In the undifferentiated state, H9c2 cells tend to be highly proliferative relying on glycolysis rather than mitochondrial oxidative phosphorylation^15^. Such aerobically poised cells can demonstrate resistance to toxic agents that target mitochondria^16^. Ceramide is a central lipid component of sphingolipid structure that is biosynthesized by three pathways, which include de novo synthesis from palmitoyl-CoA and serine, hydrolysis of sphingomyelin or a salvage pathway^17^. It is an important lipid mediator regulating various cellular responses like cell death, and recent evidence also suggests a role in various metabolic pathways influencing mitochondrial function^18^. In vitro data indicate production of ceramide increases in glycolytic cells but decreases in cells with developed OXPHOS^19^. Previous data indicate undifferentiated H9c2 cells are susceptible to DHA-induced cell death in a concentration-dependent manner which does not occur in primary neonatal cardiomyocytes^20^. Furthermore, 19,20-EDP was demonstrated to cause cytotoxicity in undifferentiated H9c2 cells correlating with de novo synthesis of intracellular ceramide^7^. While ceramide is known to induce cell death in tumor cells, the mechanisms involved in DHA-mediated events associated with a metabolic state remain unclear. In the present study, we investigated the effects of DHA and 19,20-EDP in undifferentiated H9c2 cells cultured under conditions triggering glycolytic or oxidative phosphorylation-mediated metabolism.

## Results

### Culturing non-differentiated H9c2 cells in low glucose media shifts cellular metabolism toward OXPHOS

**Fig. 1:**
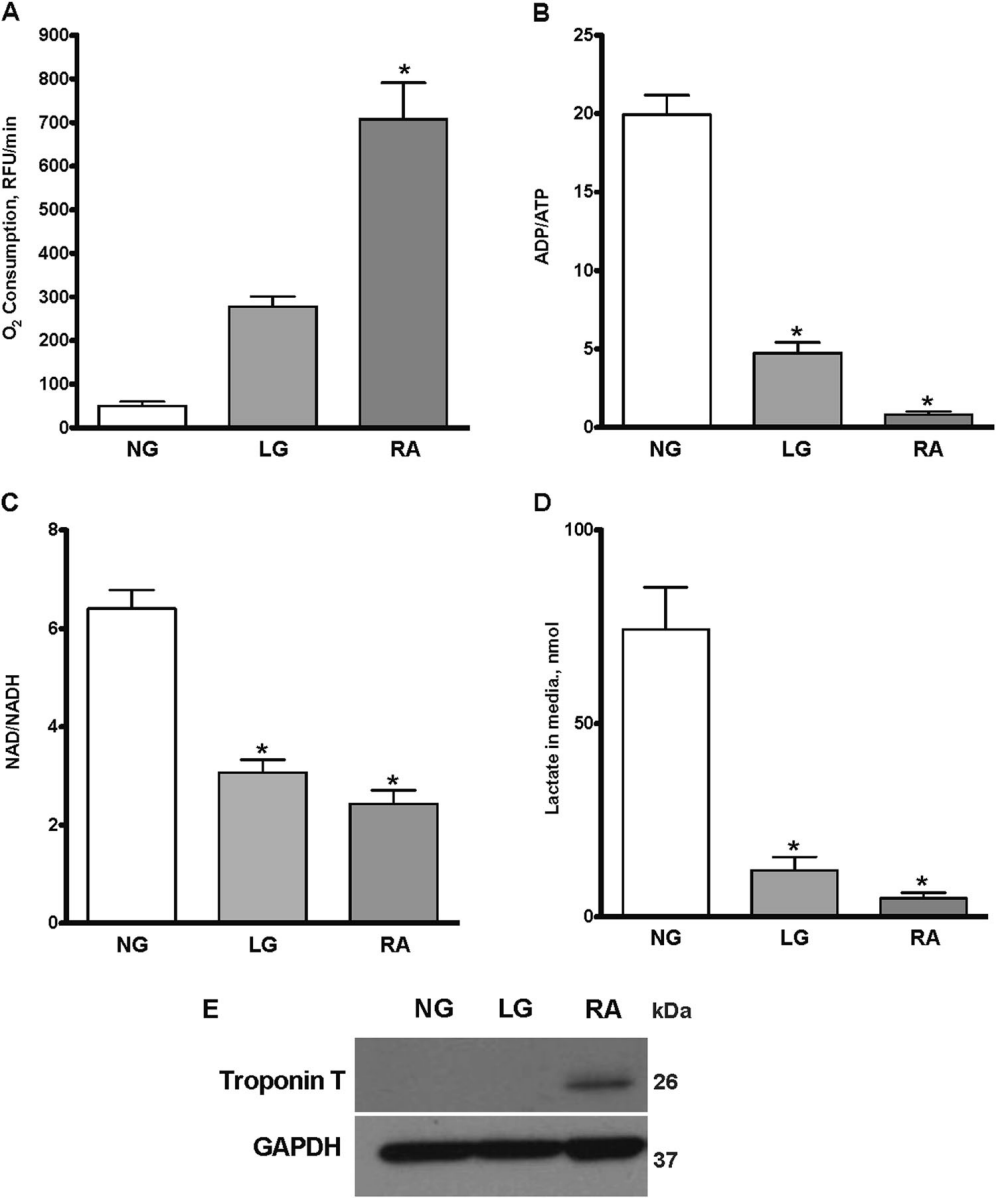
Characterization of H9c2 cells. H9c2 cells were cultured in DMEM media supplemented with 10% FBS, 25 mM (NG) or 5.5 mM (LG) glucose or cultured in DMEM supplemented with 1% FBS and 10 nM retinoic acid (RA) for 2 weeks. Alterations in culture media conditions impacted **a** oxygen consumption; **b** ADP/ATP ratios; **c** NAD/NADH ratios; and **d** lactate levels. **e** Representative immunoblot demonstrating increased expression of Troponin T in differentiated H9c2 cells incubated with 1%FBS and RA. Values are expressed as mean ± SEM (*n* = 3 repeat experiments). *P *<* 0.05 vs. NG

H9c2 cells are normally cultured in media containing 25 mM glucose, and as such they primarily utilize glycolysis for adenosine triphosphate (ATP) generation. In contrast, H9c2 cells grown in galactose or low glucose (5.5 mM) rely on mitochondrial oxidative phosphorylation (OXPHOS) to meet their energy requirements. We first demonstrated that total oxygen consumption of H9c2 cells grown with 25 mM glucose was less than one-fifth of the oxygen consumed when cells were grown in 5.5 mM glucose condition (Fig. 1a). These data suggest changing the cell culture conditions from 25 mM to 5.5 mM glucose shifted the energy metabolism in the undifferentiated cells from glycolysis to OXPHOS, which was reflected in the significant change in ATP production and increased Nicotinamide adenine dinucleotide/Nicotinamide adenine dinucleotide hydrogen NAD/NADH ratio (Fig. 1b, c). Also, we detected a significantly higher lactate level in media with 25 mM glucose, which further reflects higher glycolytic activity (Fig. 1d). Next, we assessed mitochondrial respiration in permeabilized cells to determine the respiration control ratio (RCR), which is the ratio between basal and adenosine diphosphate (ADP)-stimulated respiration. H9c2 cells grown in 25 mM glucose media had an RCR of 1.44 ± 0.18, while cells cultivated in 5.5 mM glucose media had RCR up to 6.9 ± 0.87, thus, demonstrating that there was a shift from glycolysis to OXPHOS in H9c2 cells grown in 5.5 mM glucose media.

Adding retinoic acid (RA, 10 nM) treatment to H9c2 cells cultured in Dulbecco's modified Eagle's medium (DMEM) with 1% fetal bovine serum (FBS) serum for 2 weeks has been shown to stimulate differentiation to a cardiac-specific phenotype^15^. We compared our cell model in non-differentiated cells of shifting metabolism from a primarily glycolytic metabolism to OXPHOS to differentiated cells. Neither 25 mM nor 5.5 mM cultured cells expressed troponin T, indicating our cells remained in the non-differentiated state as compared to cells treated with RA for 14 days in 1% FBS (Fig. 1e). We observed a marked difference in oxygen consumption, ADP/ATP, NAD/NADH and lactate levels between 25 mM, 5.5 mM and differentiated cells (Fig. 1a–d). Our primary goal was to investigate the impact of altered metabolism in non-differentiated cells, and hence we utilized these cells for the remaining experiments.

### DHA- and 19,20-EDP-induced cell cytotoxicity in non-differentiated H9c2 cells

**Fig. 2:**
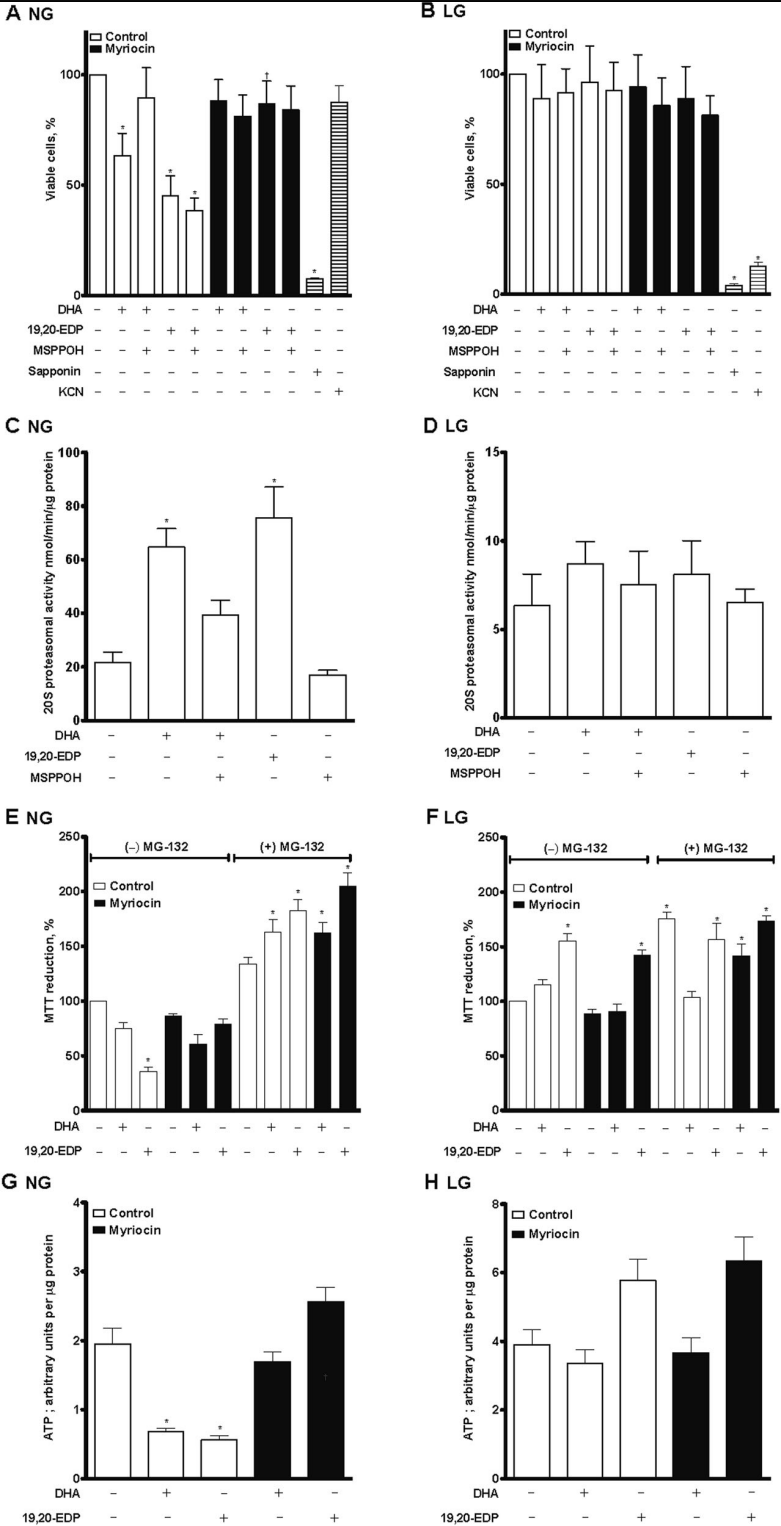
DHA and 19,20-EDP-induced cytotoxicity is attenuated by an inhibitor of de novo ceramide synthesis under glycolytic conditions. H9c2 cells were cultured in either 25 mM (NG) or 5.5 mM (LG) glucose-containing media. Differential effects of DHA and 19,20-EDP on **a**, **b** cell viability; **c**, **d** 20S proteasomal activity; and **e**, **f** MTT activity. **g**, **h** Alterations in ATP levels following incubation with DHA or 19,20-EDP. Cells were treated with or without DHA (100 μM), 19,20-EDP (1 μM), MSPPOH (50 μM), myriocin (1 μM), saponin (10 mg/mL) and/or KCN (10 μM) for 24 h. Values are expressed as mean ± SEM (*n* = 3). *P < 0.05 vs. untreated control, ^†^*P* < 0.05 vs. 19,20-EDP alone

Our previous data demonstrated that H9c2 cells cultured in 25 mM glucose were susceptible DHA-mediated cell death^20,21^. In the current study, we investigated the effect of DHA (100 µM) and its CYP epoxygenase metabolite, 19,20-EDP (1 µM), on viability of H9c2 cells grown in either 25 mM (NG) or 5.5 mM (LG) glucose-containing media (Fig. 2a, b). Consistently, both DHA and 19,20-EDP treatment markedly reduced cell viability in cells cultured in 25 mM glucose. Co-treatment of DHA with a CYP epoxygenase inhibitor (MSPPOH) prevented cell death suggesting it is the metabolite 19,20-EDP (Fig. 2a). Interestingly, neither DHA nor 19,20-EDP had any significant effect on the cell viability under low (5.5 mM) glucose conditions (Fig. 2b). Treatment with myriocin (1 µM), a potent inhibitor of serine palmitoyl transferase that inhibits the de novo synthesis of ceramide, completely blocked both DHA- and 19,20-EDP-induced cell death under 25 mM glucose conditions (Fig. 2a). Potassium cyanide (KCN), an inhibitor of mitochondrial electron transport chain complex IV, had no effect on the cell viability under 25 mM glucose conditions, consistent with a glycolytic metabolic phenotype. Conversely, KCN treatment to cells cultured under 5.5 mM resulted in an almost complete loss of viability, which is expected in cells with an OXPHOS metabolic profile. Both cell cultures were susceptible to a non-metabolic toxicant saponin (Fig. 2b).

The proteasome is a large complex type protease that degrades ubiquitinated proteins, consisting of a catalytic 20S core and two 20S regulatory subunits. Proteasomes are necessary for maintaining cell proliferation and homeostasis by regulating proteolysis of ubiquitinated and damaged proteins^22^. We measured 20S total proteasome activity following DHA or 19,20-EDP treatment under 25 mM glucose conditions to estimate the total protein degradation/turnover rate as an indication of cellular injury. We observed a significant increase in 20S total protease activity in both DHA- and 19,20-EDP-treated cells (Fig. 2c). Conversely, neither DHA nor 19,20-EDP affected total proteasome activity under 5.5 mM glucose conditions (Fig. 2d). The MTT (3-(4,5-dimethylthiazol-2-yl)-2,5-diphenyltetrazolium bromide) reduction assay can provide insight into impairment of mitochondrial oxidative activity. Our data demonstrated DHA and 19,20-EDP caused a marked decrease in activity, suggesting impaired mitochondrial function, (Fig. 2e), which was inhibited with myriocin. Interestingly, 19,20-EDP caused a significant increase in MTT reduction in H9c2 cells when treated under low glucose conditions (Fig. 2f). The presence of MG-132, a cell-permeable potent proteasome inhibitor, in both normal and low glucose conditions resulted in a significant increase in MTT activity; moreover, MG-132 blocked the effect of DHA and 19,20-EDP (Fig. 2e, f).

### DHA and 19,20-EDP cause ceramide accumulation under high glucose conditions

Since myriocin completely inhibited both DHA- and 19,20-EDP-induced cell death under normal glucose conditions (Fig. 2a), we next examined the effect of DHA and 19,20-EDP treatment toward cellular ceramide levels. The basal ceramide content in subcellular lysosomal/mitochondrial fractions isolated from cells cultured under 25 mM glucose conditions was much lower than membrane fractions (Table 1). Under 25 mM glucose conditions, both DHA and 19,20-EDP significantly increased the amount of ceramide found in membrane fractions. However, both DHA and 19,20-EDP treatment resulted in significant increases of ceramide content in lysosomal and mitochondrial fractions. Furthermore, the 19,20-EDP-induced ceramide accumulation in membrane and lysosomal/mitochondrial fractions was completely inhibited by myriocin. Cells cultured under low glucose conditions demonstrated lower basal ceramide content in both fractions. In contrast, DHA and 19,20-EDP only caused a marginal shift in ceramide levels. These results suggest DHA- and 19,20-EDP-induced cytotoxicity correlated with marked increases in movement of ceramide accumulation to lysosomal/mitochondrial fractions when cells were cultured under 25 mM glucose conditions.

**Table. 1:** De novo ceramide synthesis in H9c2 cells

	Crude membrane		Lysosome/mitochondria	
	NG	LG	NG	LG
Control	32.81 ± 1.9	14.27 ± 0.62	1.45 ± 1.27	1.14 ± 0.79
DHA	54.26 ± 1.73*	20.85 ± 0.87	470.73 ± 15.94*	0.25 ± 0.09
19,20-EDP	50.50 ± 4.63*	16.44 ± 0.52	601.73 ± 23.9*	0.40 ± 0.21
19,20-EDP+myriocin	18.23 ± 7.57^†^	11.78 ± 0.24	64.20 ± 63.85^†^	0.37 ± 0.22

LC/MS analysis was employed to measure ceramide levels in H9c2 cells cultured in either normal (25 mM) or low (5.5 mM) glucose-containing media

Ceramide content in crude membrane and lysosomal/mitochondria fractions were assessed. Cells were treated with or without DHA (100 μΜ), 19,20-EDP (1  μΜ) and myriocin (1  μΜ) for 24  h

Values are expressed as mean ± SEM (*n* = 4)

*P  < 0.05 vs. control

^†^*P* < 0.05 vs. 19.20-EDP (NG)

### DHA and 19,20-EDP suppress mitochondrial function in H9c2 cells cultured under 25 mM glucose conditions

**Fig. 3:**
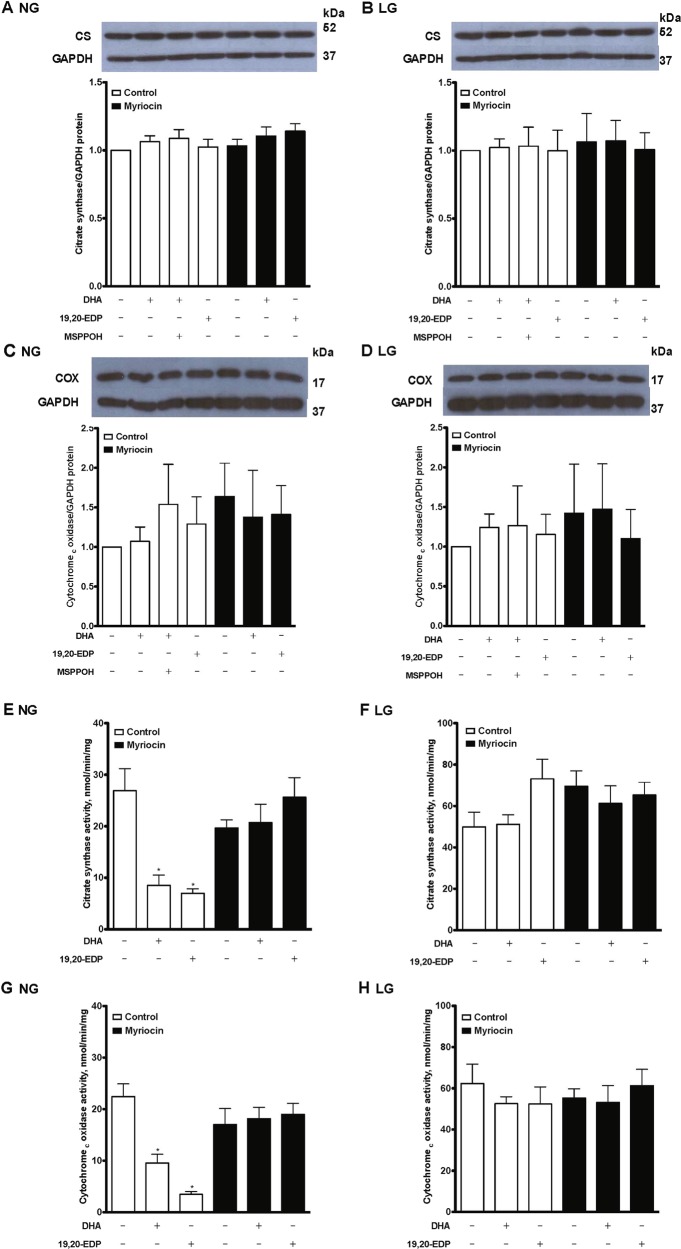
DHA and 19,20-EDP inhibit mitochondrial enzyme activities under glycolytic conditions. H9c2 cells were cultured in either 25 mM (NG) or 5.5 mM (LG) glucose-containing media. H9c2 cells were treated with or without DHA (100 μM), 19,20-EDP (1 μM), MSPPOH (50 μM) and/or myriocin (1 μM) for 24 h. Representative immunoblots and histograms of relative densities of **a**, **b** citrate synthase and **c**, **d** cytochrome *c* oxidase protein expression. Enzyme activities of **e**, **f** citrate synthase and **g**, **h** cytochrome *c* oxidase. Values are expressed as mean ± SEM (*n* = 3). *P < 0.05 vs. untreated control, *P* < 0.05 vs. 19,20-EDP alone

To define the impact of DHA and 19,20-EDP on mitochondria, we first evaluated the expression and activity of key enzymes. Under both normal and low glucose cell culture conditions, neither DHA nor 19,20-EDP had any effect on citrate synthase and cytochrome *c* oxidase protein expressions (Fig. 3a–d). However, they significantly inhibited the catalytic activities when cultured with 25 mM glucose conditions (Fig. 3e, g) and myriocin treatment significantly reversed the DHA- and 19,20-EDP-induced inhibitory effect. There is no effect on enzyme activities under low glucose condition (Fig. 3f, h).

### DHA and 19,20-EDP cause lysosome-dependent cell death under high glucose conditions

**Fig. 4:**
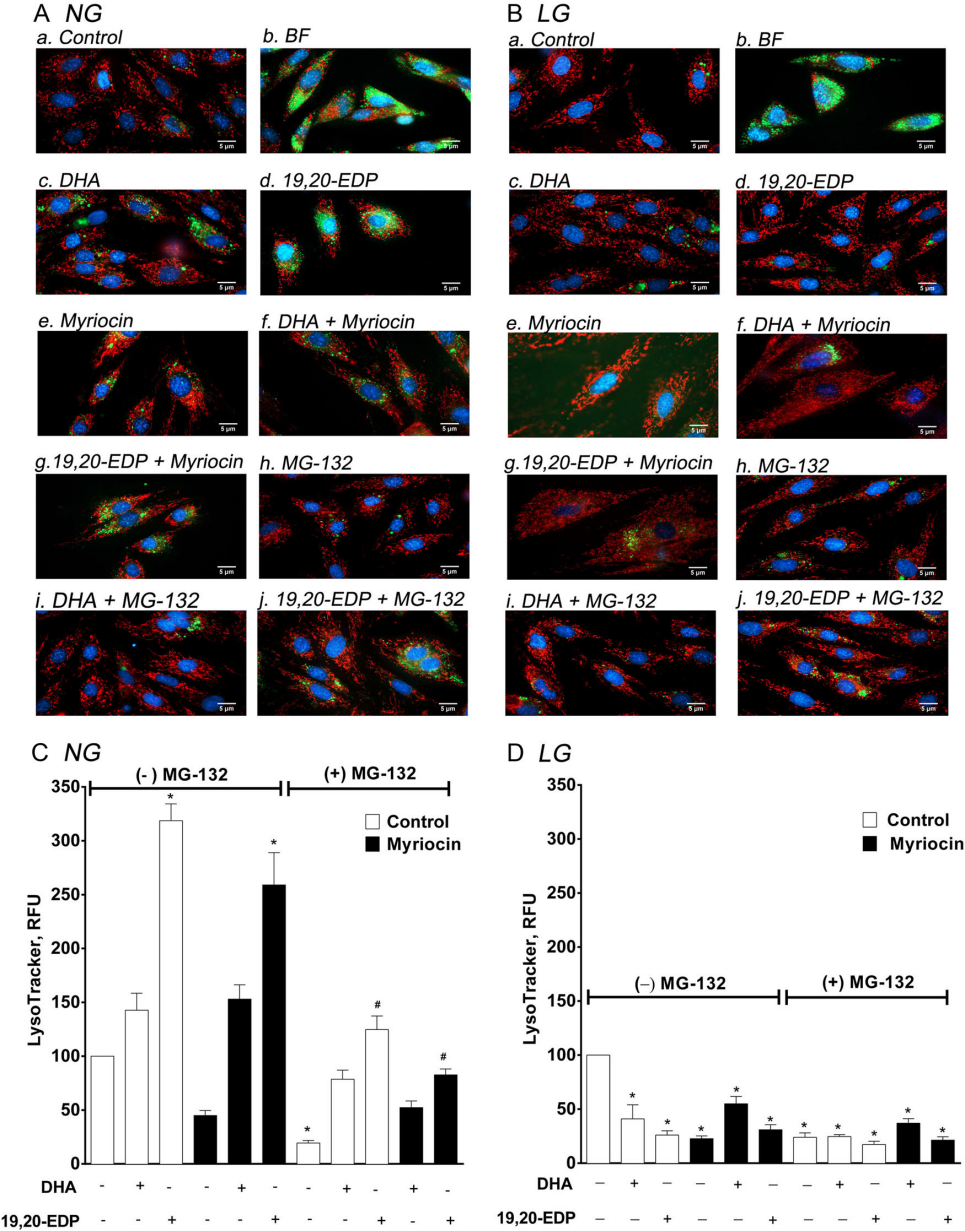
DHA and 19,20-EDP induced lysosomal formation and activity under glycolytic conditions. H9c2 cells were cultured in either 25 mM (NG) or 5.5 mM (LG) glucose-containing media. H9c2 cells were treated with or without DHA (100 μM), 19,20-EDP (1 μM), MSPPOH (50 μM), bafilomycin (1 μM), myriocin (1 μM) and/or MG1-32 (1 μM) for 24 h and then stained with mitochondrial dye, TMRE (100 nm, red), lysosomal dye, LysoTracker (10 nm, green) and nuclear dye, Hoechest33342 (1 mM, blue). Representative images of mitochondrial and lysosomal organelles in H9c2 cells cultured in **a** 25 mM (NG) or **b** 5.5 mM (LG) glucose media. Histograms representing the relative quantification of lysosomal expression in H9c2 cells cultured in **c** 25 mM (NG) or **d** 5.5 mM (LG) glucose media. Values are expressed as mean ± SEM (*n* = 3). *P < 0.05 vs. untreated control, ^#^*P* < 0.05 vs. respective group untreated MG132

Lysosomes are unique membrane-bound organelles found in the cytosol that contain degradative hydrolytic enzymes involved in digestion of many biomolecules. Lysosomal-dependent cell death has been classified as a pattern initiated by perturbations of intracellular homeostasis and the permeabilization of lysosomal membranes releasing proteases. In order to investigate the role of lysosomes we used a fluorescent LysoTracker Green dye to visualize the organelles under an epifluorescent microscope (Fig. 4A, B), as well as quantify the intensity with a fluorescent-based plate reader assay (Fig. 4C, D). Treating H9c2 cells cultured in either 25 mM or 5.5 mM glucose media with bafilomycin, as a positive control promoting acidification and maturation of lysosomes, resulted in marked increase lysosome formation (Fig. 4Ab, Bb, c). Interestingly, both DHA and 19,20-EDP increased the intensity of LysoTracker Green fluorescence (Fig. 4Ac, Ad) compared with control (Fig. 4Aa, C) under normal glucose conditions, while inhibiting ceramide synthesis with myriocin inhibited 19,20-EDP-increased intensity (Fig. 4Ag, C). DHA- and 19,20-EDP-induced intensity was reduced by MG-132 treatment (Fig. 4Ai, Aj, C). Conversely, addition of DHA or 19,20-EDP to H9c2 cells incubated in low glucose conditions had the opposite effect with a significantly decreased level of lysosomal intensity (Fig. 4B, D).

## Discussion

In the current study we demonstrate that DHA and 19,20-EDP selectively induce cell death in non-differentiated H9c2 cells with a developed glycolytic profile. It is well documented that CYP epoxygenases catalyze the enzymatic transformation of PUFA into various potent epoxylipid including omega-3 epoxides of DHA referred as EDPs. Recent evidence suggests that EDPs are involved in regulating cellular processes such as inflammation, angiogenesis and cell death; however, our understanding of how EDPs regulate these effects remains significantly limited. Previously, we have demonstrated immortalized H9c2 cells were prone to cytotoxicity caused by DHA and 19,20-EDP. The current study demonstrated the cytotoxicity of EDPs was dependent upon the metabolic profile of the cells.

Interestingly, an increased de novo synthesis of ceramide occurs following treatment DHA and 19,20-EDP, with a corresponding accumulation in intracellular membrane fractions including mitochondria and lysosomes, while H9c2 cells driven to demonstrate an OXPHOS phenotype were not susceptible to DHA- or 19,20-EDP-mediated cell death, or changes in intracellular ceramide production. Altered levels of endogenous lipids, such as ceramide, are well known to be involved in both cell survival and cell death processes. Together, our data suggest a novel cell death mechanism for DHA and 19,20-EDP lipids, which occurs in non-differentiated glycolytic cells. Noteworthy, during the last decade, studies dedicated to biological effects of DHA have largely ignored the roles of CYP-derived metabolites. In this study, we demonstrated that DHA produced cytotoxic effect in glycolytic H9c2 cells similar to what was observed with 19,20-EDP. Furthermore, formation of endogenous EDPs has an essential role in DHA- associated injury of glycolytic H9c2 cells. Since cytotoxic effects of DHA toward glycolytic H9c2 cells were strongly decreased when MSPPOH, a CYP epoxygenase inhibitor, was added, we presume that observed effects were associated with EDPs endogenously produced from DHA. One of the main metabolic divergences between quiescent and proliferating cells is the pathway responsible for ATP synthesis. In non-cancerous cells with hallmarks of primary lines, ATP is usually synthesized during OXPHOS, whereas cancerous and highly proliferative cells preferentially develop glycolysis for ATP synthesis and employ mitochondria solely to sustain redox potential required to reduce NADH for glycolysis. This is known as the Warburg effect that results in formation of a pool of aberrant mitochondria with poor functional activity^23,24^.

While our previous research correlated DHA-mediated cell death in H9c2 cells with ceramide production, the current data demonstrated unique mechanisms^20^. The sphingolipid, ceramide, has important roles in cellular membrane structure and is an integral component of the sphingomyelin cycle acting as an essential second messenger in the intracellular propagation of physiological, pharmacological and environmental signals in normal and cancerous cells^25^. Ceramide elicits many cell-stress responses including apoptosis, senescence, inflammation, mitochondrial dysfunction and altered cellular metabolism^17,26,27^. The hydrophobic nature of ceramide limits its accumulation to membranous regions within the cell where it may influence protein distribution and function. In this regard, cytosolic concentrations of ceramide are negligible. While the majority of studies evaluating the effects of an elevation in cellular ceramide have focused on its ability to induce apoptosis^19^, several other studies have also described ceramide to be necessary for the induction of senescence, cell–cell interactions, death receptor clustering and autophagy^25,28–33^. Collectively, these studies emphasize that ceramide is capable of modulating several biochemical pathways^17,25,28–34^. Strategies that pharmacologically or genetically decrease ceramide have beneficial effects in reversing insulin resistance, preventing apoptosis of pancreatic β-cells and cardiomyocytes^28–30^.

Accumulation of ceramide has been linked to major perturbations in cell metabolism resulting in apoptosis, necroptosis and lethal autophagy including mitophagy^15,28,31,33^. Ceramide accumulation in mitochondria can lead to stress-induced mitochondrial fragmentation and decreased ATP production through disruption of electron transport chain and induction of injury, resulting in a pool of dysfunctional mitochondria capable of promoting cell death^31,32^. We found that both DHA and 19,20-EDP induced ceramide accumulation in mitochondrial and lysosomal fractions, which was diminished in the presence of myriocin. Moreover, the increased de novo synthesis of ceramide and its accumulation in subcellular membrane fractions occurred under glycolytic conditions and requires increased NADH reduction, which was markedly reduced under OXPHOS^35^. The increased production of ceramide correlated with elevated lysosomal activity and was reversed by the proteasome inhibitor MG-132, which additionally support the role of ceramide formed in glycolytic conditions in various types of cell death^36^. Detection of lysosomal-mediated cell death originally was difficult, as lysosomal ultrastructure often appears intact in apoptotic cells analyzed by electron microscopy^37^. Partial lysosomal membrane permeabilization can occur early in many death paradigms, which triggers proteasomal cell death^38^. Interestingly, increases in ceramide levels have been shown to lower the integrity of lysosomal membranes and hinders their fusion with other intracellular vesicles including mitochondria^38^. In the current study, accumulation of ceramide in lysosomal fractions following treatment with DHA or 19,20-EDP may have contributed to destabilization of lysosomal membranes and the subsequent increase in proteasomal and lysosomal activities. Early studies using compounds directly targeting the integrity of lysosomal membranes provided evidence for their role in programmed cell death^39–41^. Moreover, inhibitors of lysosomal function would be more toxic to cancer and transformed cells than normal cell lines^37^. A quantitative relationship between the amount of lysosomal rupture and the mode of cell death has been suggested to explain the widely different morphological outcomes following lysosomal membrane permeabilization^42^. According to this model, low stress intensities trigger a limited release of lysosomal contents to the cytoplasm followed by cell death, while high intensity stress factors lead to a generalized lysosomal rupture and rapid cellular necrosis. It is entirely possible that DHA and 19,20-EDP induced accumulation of ceramide in H9c2 cells with glycolytic profile, which instigated cell death. Remarkably, co-treatment with MG-132, an inhibitor of lysosomal proteases, decreased toxicity of DHA and 19,20-EDP.

In conclusion, we report a novel cell death mechanism for DHA and its epoxylipid, 19,20-EDP, in non-differentiated glycolytic H9c2 cells. Our study suggests these lipid mediators induce a ceramide-mediated process that increases lysosomal-proteasome activity impacting mitochondria function leading to cell death. An increased production of ceramide coupled with increased lysosomal membrane permeability in glycolytic cells causes a destabilization of mitochondria resulting in cell death. The shift in cellular metabolic profile from glycolytic to OXPHOS highlights a novel toxicity of DHA and 19, 20-EDP toward H9c2 cells with a glycolytic profile.

## Materials and methods

### Cell culture and viability assay

H9c2 cardiac cells were obtained from ATCC. The cells were cultured in DMEM medium containing either 25 mM (normal glucose) or 5.5 mM glucose (low glucose) and supplemented with 10% FBS and 1% penicillin/streptomycin. Differentiation of H9c2 myoblasts into myotubes was performed in DMEM supplemented with 1% FBS, 1% penicillin/streptomycin and 10 nM retinoic acid for 2 weeks. Cell viability was assessed using a commercially available kit from Promega based on luminescent assay of intracellular proteases. Another approach to measure metabolically active cells was MTT test performed as previously described^43^. The intensity of reduction of MTT to formazan crystals by mitochondrial dehydrogenases positively correlates with the overall activity of oxidative metabolism. Optical density of dimethyl sulfoxide extracted from formazan was measured spectrophotometrically at 595 nm.

### LC/MS measurement of different pools of intracellular ceramide

The extraction of ceramide from H9c2 cells was performed in accordance to a published protocol^44^. Cells were cultured in 175 cm^2^ flasks and then harvested and subjected to subcellular fractionation. Cells were centrifuged at 700 × *g* for 10 min and then rinsed with cold phosphate-buffered saline (PBS) and centrifuged again at 700 × *g* for 10 min, 4 °C. The resulting pellet was termed and used as a crude membrane fraction. For the enrichment of heavy membrane fraction containing light and heavy mitochondria as well as lysosomes, the remaining supernatant was centrifuged at 10,000 × *g* for 20 min, 4 °C. Then, 300 mL of a 0.4% NaCl solution and 1 mL of a chloroform–methanol–HCl 1 N (100:100:1, v/v/v) mixture were added to the samples. Following this, the samples were vortexed at 1000 rpm (room temperature) for 20 min. Ceramide content in the samples was analyzed using Waters ZQ 4000 Mass Spectrometer coupled to a Water 2795 Separations Module. A previously described liquid chromatography/mass spectrometry (LC/MS) method was utilized with modifications^45^. Briefly, ceramide as well as the internal standard, 4-methoxybenzophenone, were resolved using a reverse-phase C18 column (Alltima HP, 150 × 2.1 mm) at 35 °C by the isocratic elution of methanol–tetrahydrofuran–water–acetic acid (80:10:10:0.2, v/v/v/v) as the mobile phase. Ceramide and 4-methoxybenzophenone were detected using a single ion recording under positive-ion mode at *m/z* = 342.4 and 213.1, respectively. The cone voltage was 25 V and 15 V for ceramide and 4-methoxybenzophenone, respectively, and capillary voltage of 4.0 kV. During the acquisition of the data, the mass spectrometer was maintained at a source and desolation temperature of 120 °C and 275 °C, respectively. The resulting values were then calculated using a ceramide standard curve and expressed as mg weight per mg of cellular mass, and then further normalized to account for the fold change between wet and dry cellular mass for quantification purposes.

### Assessment of mitochondrial function

Cellular ATP was determined using a kit based on fluorescent assay (Abcam or Sigma-Aldrich). Citrate synthase (CS) and cytochrome *c* oxidase enzymatic activities were assayed spectrophotometrically in cell lysates as previously described^46,43^. Rapid assessment of oxygen consumption was performed using a kit based on detection of oxygen with fluorescent trap (Abcam). NAD/NADH ratio was assessed using a kit from Promega. Mitochondrial respiration was measured in saponin permeabilized HL-1 cells using Clark oxygen electrode connected to Oxygraph Plus recorder (Hansatech Instruments Ltd, Norfolk, England). Respiration rates were measured at 30 °C before and after addition of 2 mM ADP with 5 mM malate and 10 mM glutamate as substrates. RCR was calculated as the ratio between basal and ADP-stimulated respiration rates^7^.

### Immunoblotting

Samples were subjected to western blot analysis as previously described^7,20^. Briefly, 25 μg of protein from H9c2 lysates was probed on a 12% SDS-polyacrylamide gel. Membranes were washed four times with Tris-buffered saline with Tween-20 buffer and then incubated with primary antibodies, CS (1:000, Abcam., Cambridge, UK, Cat No: ab129095), cytochrome *c* oxidase (COX IV) (1:1,000, Cell Signaling Tech., Inc., Whitby, ON, Cat No: 4850) and GAPDH (1:5000, Cell Signaling Tech., Inc., Whitby, ON, Cat No: 5174S), secondary antibodies were used as 1:5000 dilution. Relative band intensity to control was measured using Image J software (NIH, USA).

### Assessment of lysosomal activity

Lysosomal activity was measured and quantitated using LysoTracker Green DND-26 which specifically stains acidic structures in live cells. H9c2 cells were treated with LysoTracker Green DND-26 added to a final concentration of 10 nM for 1 h. The cells were rinsed with PBS and fluorescence was measured with excitation/emission 504/511 nm. The intensity of fluorescence reflected the active pool of lysosomes. MG-132, which acts as a potent cell-permeable inhibitor of lysosomal activity, was added to a final concentration of 1 µM where indicated.

### Microscopy

H9c2 cells were grown on glass-bottom 35 mm dishes suitable for fluorescent microscopy (MatTek) and treated with indicated reagents. Mitochondria were stained with TMRE (100 nM) and nucleus were stained with Hoechest33342 (1 mM). Acidic organelles were stained with LysoTracker Green DND-26 (10 nM). The dishes were placed in a micro incubator installed on the objective stage of a Zeiss Axio Observer epifluorescence microscope. The image system consisted of Zeiss Axio Observer Z1 inverted epifluorescence microscope equipped with a LED lamp, 63× oil immersion objective lens and cooled CCD camera.

### Statistical analysis

Values were expressed as mean ± standard error of mean. Statistical significance was determined using one-way analysis of variance. To determine whether significant differences exist between the groups, Bonferonni post-hoc test was performed. Values were considered significant as *p* < 0.05.
